# Can Keratin Scaffolds be used for Creating Three-dimensional Cell Cultures?

**DOI:** 10.1515/med-2020-0031

**Published:** 2020-04-03

**Authors:** Marta Bochynska-Czyz, Patrycja Redkiewicz, Hanna Kozlowska, Joanna Matalinska, Marek Konop, Piotr Kosson

**Affiliations:** 1 Toxicology Research Laboratory, Mossakowski Medical Research Centre Polish Academy of Sciences, 02-106 Warsaw, 5 Pawinskiego Street, Poland; 2 Department of Neuropeptides, Mossakowski Medical Research Centre Polish Academy of Science, 02-106 Warsaw, 5 Pawinskiego Street, Poland; 3 Laboratory of Advanced Microscopy Techniques, Mossakowski Medical Research Centre Polish Academy of Sciences, 02-106 Warsaw, 5 Pawinskiego Street, Poland

**Keywords:** 3D keratin scaffolds, KAP, fur, cell culturing

## Abstract

Three-dimensional (3D) cell cultures were created with the use of fur keratin associated proteins (F-KAPs) as scaffolds. The procedure of preparation F-KAP involves combinations of chemical activation and enzymatic digestion. The best result in porosity and heterogeneity of F-KAP surface was received during pepsin digestion. The F-KAP had a stable structure, no changes were observed after heat treatment, shaking and washing. The 0.15-0.5 mm fraction had positive effect for formation of 3D scaffolds and cell culturing. Living rat mesenchymal cells on the F-KAP with no abnormal morphology were observed by SEM during 32 days of cell culturing.

## 1 Introduction

The regeneration of tissue at the site of injury or wounds caused by diseases such as diabetes is a challenging task in the field of biomedical science. Regenerative medicine and tissue engineering need complementary key ingredients, like biologically compatible scaffolds that can be easily adopted by the body system without harm, and suitable cells including various stem cells that effectively replace the damaged tissues without adverse consequences. The scaffold should mimic the structure and biological function of the native extracellular matrix at the side of injury for regeneration of tissues [1]. For this purpose, many researchers have been motivated toward tissue engineering which led to the development of implantable porous scaffolds to be used for cell cultures and to regenerate the body at the damage site [2, 3, 4, 5, 6].

Protein-based biomaterials are one of the promising options for this application. They have emerged as potential candidates for many biomedical and biotechnological applications due to their ability to function as a synthetic extracellular matrix that facilitates cell-cell and cell-matrix interactions [7]. Such protein-based biomaterials have defined three-dimensional microstructures that support cellular proliferation and cell-guided formation of tissue.

Several proteins such as: collagen, albumin, gelatin, fibroin and keratin have been investigated in the development of naturally derived biomaterials [8, 9, 10]. Keratin-based materials characterized as less thermolabile proteins, with mechanical durability, intrinsic biocompatibility, biodegradability and natural abundance can be the most promising for revolutionizing the biomaterial world.

The results of our studies show that the fibrous 3D structure of keratin provides a favorable 3D microenvironment for cell cultures used in regenerative medicine.

## 2 Materials and methods

**Figure 1:**
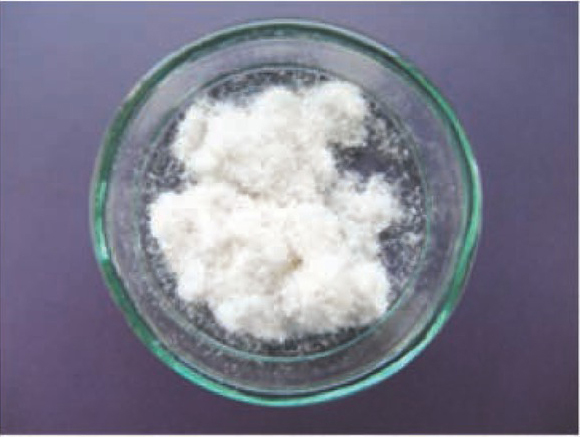
Macroscopic image of the scaffolds (F-KAPs) after enzymatic treatment.

Keratin scaffolds were prepared by using rat fur according to the reported method investigated in our laboratory by A.W Lipkowski [11,12]. Briefly, rat fur was suspended in 2% solution of NaOH for 1 hour at room temperature (21C) and occasionally mixing. Next, the material was filtered off and washed with water until reaching pH 7.0 in the eluate, and air-dried at room temperature. Then, fur were suspended in water which was acidified or alkalized to pH appropriate for tested enzyme. Three different enzymes (pepsin, papain and pancreatin) were added to the suspension, and reaction was continued at 37°C with shaking for 24 hours. Residue was filtered off and dried. Then, solid material was digested again 24 hours with pepsin, papain or pancreatin, washed with water and dried. The final product was filtered off, washed with water and dried. This product was defined as fu r keratin associated proteins (F-KAPs) preparation. Dry material was ground to the small fragments and divided according to the size of filaments into several fractions (Fig.1).

Mesenchymal stem cells from Wistar rats (rMSC, Millipore) were multiplied in order to increase their number, on a standard expansion medium: DMEM (Sigma Aldrich) supplemented with 15% FBS (Gibco) and 1% antibiotic-penicilin/streptomycin solution (Sigma). Cultures were maintained in air with 5% CO_2_ and 95% humidity at 37° C. The cells were harvested, washed with PBS (Biomed, Lublin) and seeded (5000 cells in 50 ul of medium) onto F-KAP. The scaffolds stood in cell incubator for 30-45 min. to promote cell adhesion onto F-KAP surface and were placed on 10x35 mm Petri dishes with 6 ml of cultivation medium. The medium was exchanged every 2-3 days for the first two weeks of culturing, after this time every day.

rMSC cells on F-KAP were fixed with 4% paraformaldehyde. Next, aggregates were blocked and permeabilized with 10% normal goat serum (Sigma) in 0,25% Triton X-100 for 1h at room temperature. Then, the primary antibody, anti-vimentin mouse antibody IgG (Dako), was applied for 24h at 4^o^ C. After washing 3 times with PBS, the secondary antibody, goat anti-mouse conjugated with Alexa 488 fluorochrome (Invitrogen), was applied for 1h at RT. Cell nuclei were stained with 5 uM Hoechst (Sigma) for 15 min. After final wash the aggregates were mounted in Fluorescence Mounting Medium (DAKO). To obtain detailed images of the cells, a confocal laser scanning microscope (Cell Obsever, Carl Zeiss, Oberkochen, Germany) was used. The shape and surface morphology of the scaffolds (substrates and products) and cells on scaffolds were observed by scanning electron microscope (SEM) type JEOL JSM-6490 LV (Japan).

## 3 Results and discussion

**Figure 2:**
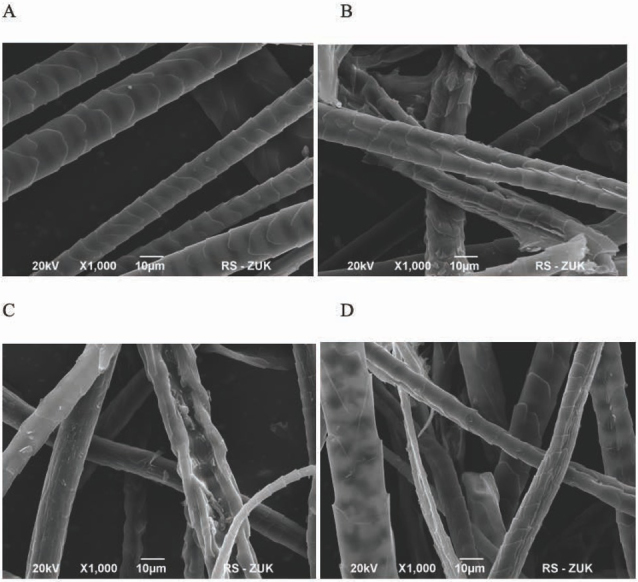
Scanning electron microscopic observation: (A) control – untreated fur fibers and fur fibers (F-KAPs) digested with (B) pepsin, (C) papain, (D) pancreatin.

**Figure 3:**
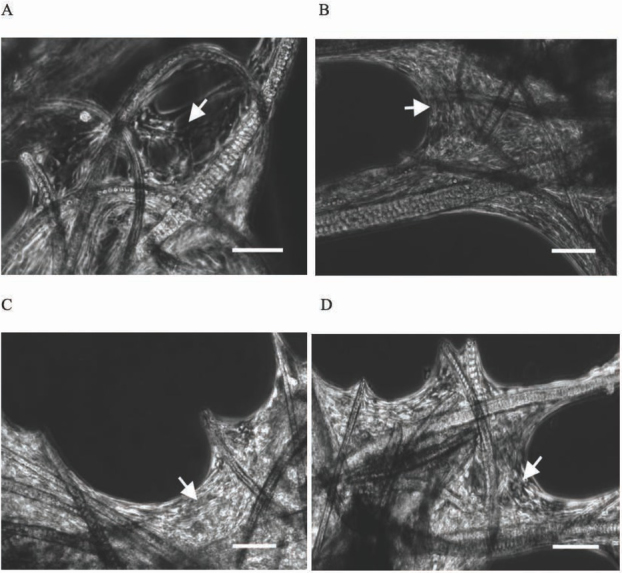
Light microscopic observation of cell growing (rMSC) on F-KAPs. (A) 14 days culturing, (B) 21 days culturing, (C) and (D) 32 days culturing, arrow shows rat mesenchymal stem cells, scale bar = 200 **μ**m.

**Figure 4:**
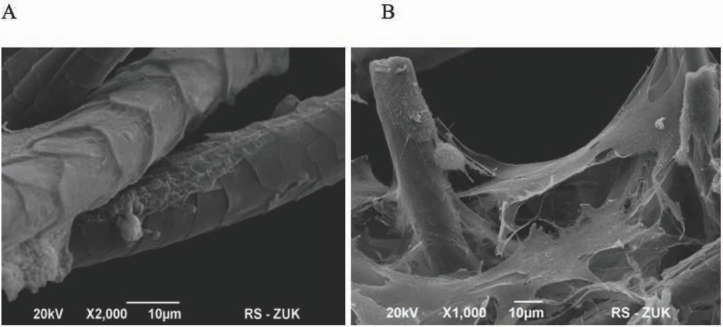
Scanning electron microscopic observation of the rMSC growth on F-KAPs digested with papain. (A) At first 7 days after seeding, cells adhered on the surface of F-KAPs scaffolds. (B) At 32 days, the cells supported by F-KAPs formed multilayer.

3D keratin scaffolds exist in various forms, for example as gels [2,3,5], sponges [8,14], films [15,16] or fibers [17, 18, 19] with the use of various preparation methods. In this paper we present the use of fiber keratin scaffolds obtained from rat fur. We observed that using appropriate digest enzyme was significant for obtaining the proper microstructure and the surface roughness. We tested three different enzymes: pepsin, pancreatin and papain (Fig. 2 A-D) and in our case the best result in porosity and heterogeneity of F-KAP surface was received during pepsin digestion (Fig. 2 B). The condition of F-KAP preparation had no unfavorable impact on the stability of scaffold structure. During the heat treatment, shaking and washing the F-KAP showed no significant changes. Simultaneously, keratin scaffolds were grounded and separated into fractions by size of keratin fibers. Scaffolds were sieved through a sieve to afford three main fractions: 0.05-0.15 mm; 0.15-0.5 mm; 0.5-1.0 mm in length and average diameter 50 um. We have observed that 0.05-0.15 mm fraction was too powdery and cells did not have the ability to attach to them and proliferate, and 0.5-1.0 mm fraction did not form the stable 3D scaffolds (data not shown). In our experiment the best results in cell culturing and positive effect on the formation 3D scaffolds had 0.15-0.5 mm fractions. The similar size of scaffolds was used also in the study of Rivet et al. [19]. Rat mesenchymal stem cells were used to assess cell ability to grow up and form the 3D colonies on rat F-KAP. Adhesion of cells on the surface of keratin scaffolds was observed after a few hours from seeding. During next 7 days of culturing cells proliferated and from day 14 cells joined to each other and new connection between cells and fibers appeared to form 3D colonies. Next, from day 21 we observed the growing up of the colonies located in different depth creating multilayer surface around the F-KAP scaffolds. These changes were observed in micro-photographs of light (Fig. 3 A-D) and scanning electron microscopy (Fig. 4 A-B).

**Figure 5:**
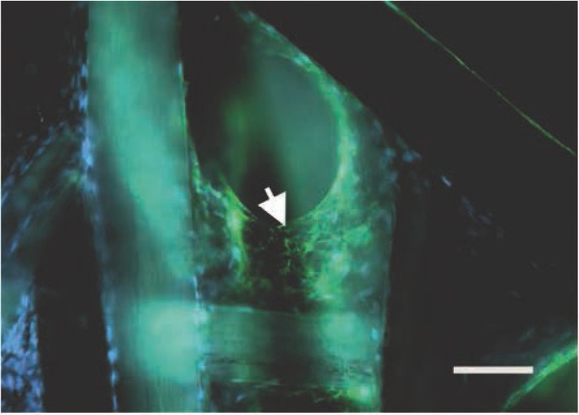
Confocal laser scanning microscope observation of the rMSC growth on F-KAPs stained with vimentin and Hoechst, arrow shows rat mesenchymal stem cells, scale bar = 200 μm.

Additionally, stained with Vimentin and Hoechst co-cultures confirmed forming 3D cell culturing on F-KAP (Fig. 5). The most visible colonies were observed in places where keratin fibers crossed each other. We were able to maintain created co-cultures in vitro for 32 days without any abnormal morphology of cells and any significant evidence of cell death.

In conclusion, our study shows that co-cultures of rat mesenchymal stem cells on F-KAP confirm the formation of 3D cell culturing on keratin scaffolds. The co-cultures can be maintained *in vitro* for several weeks without morphological changes of cells and with no observed apoptosis. The above properties and also low immunogenicity and biodegradation [20] of KAP scaffolds can be recommended as promising candidate for the application in the field of biomedical science, including tissue engineering with the prospective use in clinical applications. Additionally, it is possible to introduce different bioactive substances into KAP scaffolds which could improve and increase the functionality of the KAP scaffolds.
